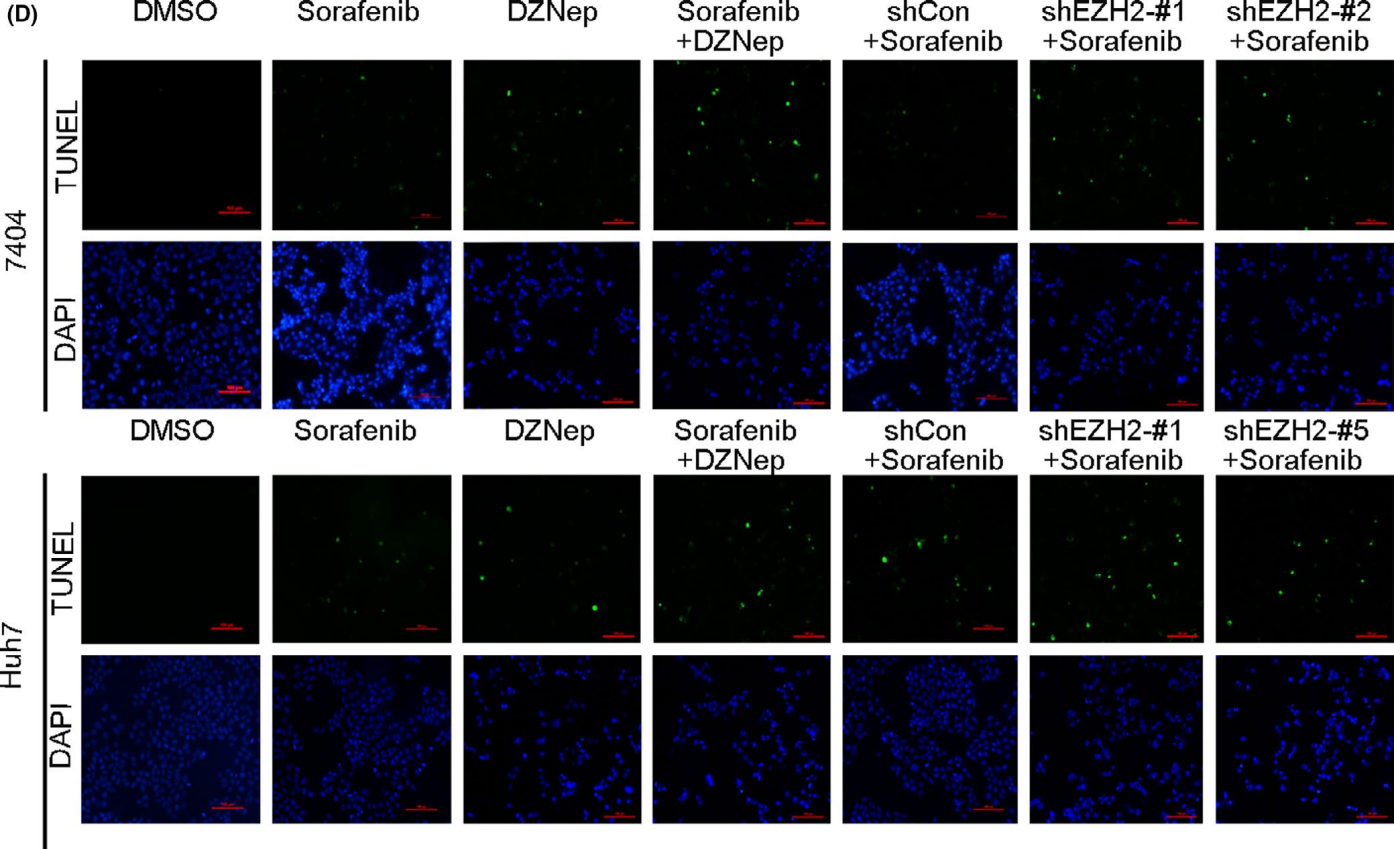# CORRECTION

**DOI:** 10.1111/cas.15203

**Published:** 2021-12-05

In an article^1^ titled “Sorafenib suppresses growth and survival of hepatoma cells by accelerating degradation of enhancer of zeste homolog 2” by Shanshan Wang, Yu Zhu, Hongyong He, Jing Liu, Le Xu, Heng Zhang, Haiou Liu, Weisi Liu, Yidong Liu, Deng Pan, Lin Chen, Qian Wu, Jiejie Xu, Jianxin Gu, the authors would like to make changes on Figure 1A, Figure 5C, and Figure 5D.

In Figure 1(A), the strips from HepG2 and Huh7 cell lines have been replaced by new strips. The correct strips in Figure 1(A) are shown below.

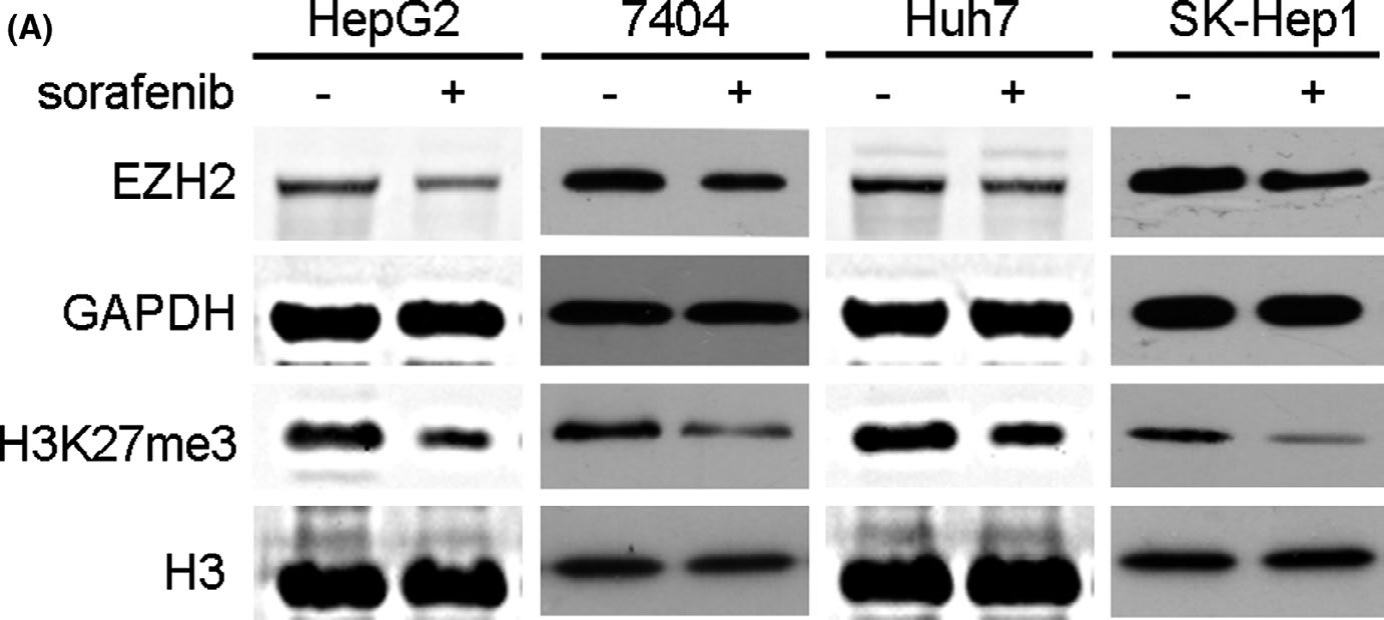

In Figure 5(C), the colony image of Sorafenib + DZNep treated Huh7 cells in colony formation assay was accidently misplaced and has been replaced by the correct image now. The correct colony image of Sorafenib + DZNep treated Huh7 cells is shown in the figure below.

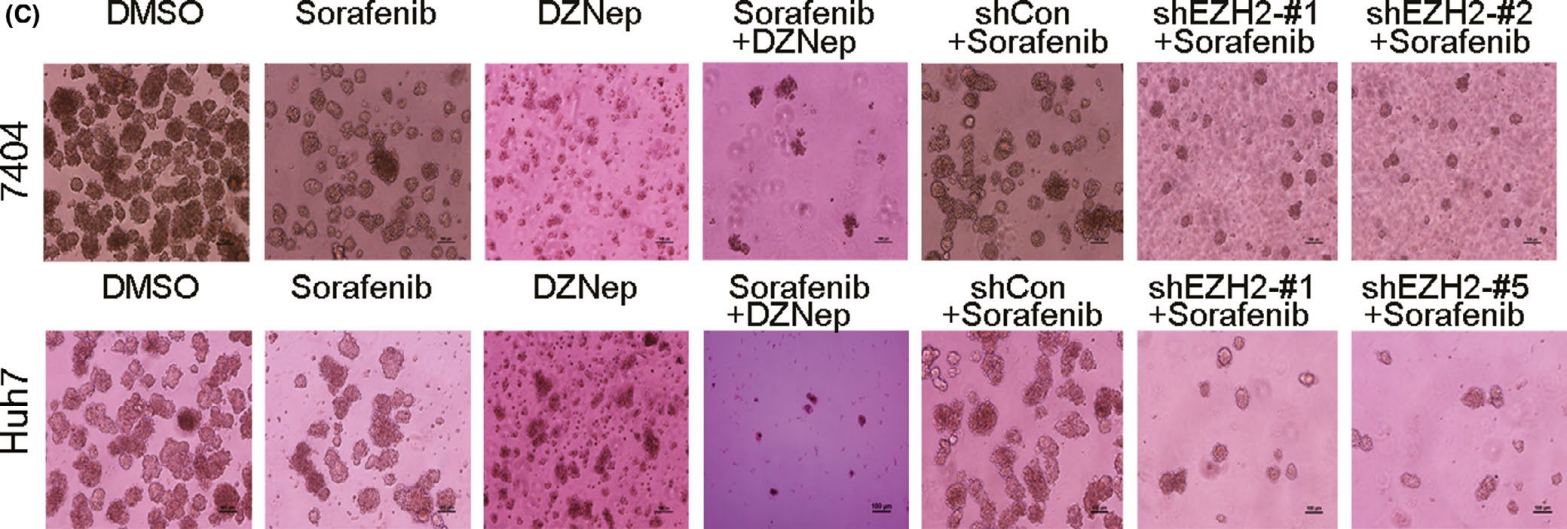

In Figure 5(D), the TUNEL staining and DAPI staining image of DMSO treated 7404 cells in TUNEL assay was accidently misplaced, and has been replaced by the correct image now. The correct the TUNEL staining and DAPI staining image of DMSO treated 7404 cells in TUNEL assay in the figure is shown below.